# Research progress on social participation of young and middle-aged stroke survivors: a narrative review

**DOI:** 10.3389/fpsyt.2026.1775209

**Published:** 2026-04-16

**Authors:** Mei He, Pan Cai

**Affiliations:** 1 The Second Affiliated Hospital of Zunyi Medical University, Zunyi, Guizhou, China; 2 The Third Affiliated Hospital of Zunyi Medical University/The First People’s Hospital of Zunyi, Zunyi, Guizhou, China

**Keywords:** narrative review, assessment tools, influencing factors, social Participation, stroke, young and middle-aged adults

## Abstract

Stroke is characterized by high morbidity, disability, and mortality, and has become the third leading cause of death worldwide. In China, stroke accounts for 39.9% of all cerebrovascular diseases, with young and middle-aged survivors (aged 40–60 years) comprising 33% of global stroke survivors in this age group and over 51.51% of all stroke cases in China. Despite significant improvements in treatment, 70–80% of survivors still lose the ability to live independently, and social participation declines to varying degrees. Social participation plays an important role in rehabilitation outcome indicators, which can reflect the overall recovery of survivors and is closely related to quality of life. Guided by the International Classification of Functioning, Disability and Health (ICF) framework, this review aims to examine the current status, assessment tools, and influencing factors of social participation among young and middle-aged stroke survivors, with the goal of informing future research and guiding clinical practice.

## 1 Introduction

**Figure 1:**
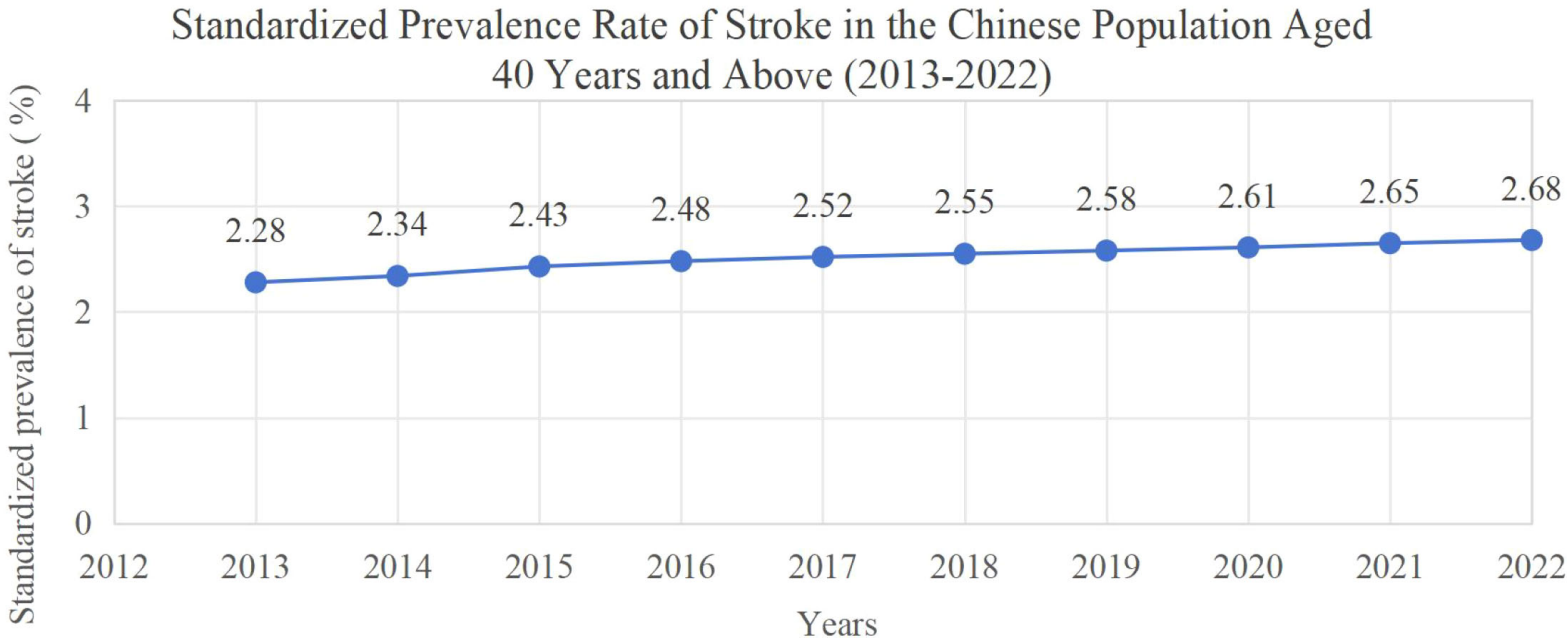
Standardized prevalence rate of stroke in the Chinese population aged 40 years and above (2013–2022). The unit is percentage (%), and the values (2.28%–2.68%) represent the real epidemiological characteristics of stroke prevalence in Chinese adults aged ≥40 years. The population includes both young and middle-aged adults (40–60 years, accounting for 51.51% of all stroke cases in this age group) and older adults (≥65 years). Data were obtained from the China National Stroke Screening and Intervention Program (CNSSIP) and the 2020–2024 China Stroke Prevention and Treatment Report.

Stroke, also known as a cerebrovascular accident, is associated with high rates of morbidity, disability, and mortality. It is currently the third leading cause of death worldwide (1). Globally, an estimated 93.8 million individuals are living with the effects of stroke (1), with approximately 3.4 million new cases reported annually in China alone (2). Approximately 75% of stroke survivors experience some level of functional impairment (e.g., motor, sensory, speech, or swallowing disorders), and about 40% go on to develop severe long-term disabilities(e.g., hemiplegia) (3). The complex, time-sensitive process of post-stroke neural repair—including axonal remodeling, neurogenesis, and angiogenesis (4)—often restricts functional recovery, leaving many survivors with long-term sequelae (5). Some may even suffer from long-term disorders of consciousness (6), placing a substantial burden on both families and society. China faces a particularly heavy burden of stroke, with a striking trend toward younger onset. As illustrated in **Figure 1**, the age-standardized prevalence of stroke among individuals aged 40 and above has exhibited a continuous upward trend from 2.28% in 2013 to 2.68% in 2022. Despite this increase of only 0.40 percentage points, combined with China’s population base aged 40 and above, it corresponds to a significant rise in the actual number of stroke patients; moreover, young and middle-aged patients (40–60 years old) account for 51.51% of all stroke cases in China, and the growth in their prevalence has important public health impacts on labor supply, family care, and medical resource allocation. Notably, this burden is heavily concentrated within the working-age population: young and middle-aged adults (40–60 years) account for 51.51% of all stroke cases in China (7), representing approximately one-third (33%) of global stroke cases within the same age group (8). With the establishment of stroke centers and wards and the development of emergency and critical care medicine, the mortality and recurrence rates of stroke survivors have been reduced. However, the disability rate among survivors continues to rise, substantially impairing their quality of life and potentially compromising long-term survival outcomes (9). For young and middle-aged individuals—who bear significant personal, familial, and societal responsibilities—post-stroke sequelae can result in physical impairments, financial strain, and psychological distress, ultimately reducing their social participation (10). Social participation is considered a key indicator of rehabilitation outcomes, as it provides a holistic measure of a patient’s functional recovery and is strongly associated with both quality of life and long-term outcomes. By establishing connections with others through social activities and exposing themselves to new information and challenges, survivors can promote the connections between neurons in the brain, which helps to delay the decline of cognitive function (11). In addition, social participation is conducive to alleviating survivors’ negative emotions such as anxiety and depression, and promoting their physical and mental health (12). However, studies have shown that individuals with multiple chronic conditions, including stroke survivors, tend to have lower levels of social participation (13), a phenomenon that is pronounced among working-age adults (40–60 years) and may impede their functional recovery (11). While research on post-stroke social participation has expanded in recent years, it has focused on validating rehabilitation interventions, analyzing determinants within the ICF framework, or exploring generic intervention strategies (14, 15); yet has overlooked the needs of young and middle-aged survivors (e.g., vocational reintegration, family caregiving). To address this gap, this narrative review aims to review the existing literature on social participation in young and middle-aged stroke survivors. Guided by the ICF framework, this paper focuses on individuals aged 40–60 years, examining the current state of their social participation, relevant assessment tools, and associated determinants. The objective is to clarify research gaps in this field and provide evidence-based implications for future clinical interventions and research design.

## 2 Concept and current situation analysis of social participation

### 2.1 Evolution of the concept of social participation

In 2001, the World Health Organization (WHO) introduced the International Classification of Functioning, Disability and Health (ICF), a framework designed to describe and measure physical and mental health and functioning. A key feature of the ICF is its emphasis on “participation”, reflecting a conceptual shift from focusing solely on physical functioning to incorporating social functioning in the evaluation of rehabilitation outcomes. As a result, “social participation” has gradually emerged as a critical outcome variable for assessing rehabilitation effectiveness and functional independence (16). However, the concept of social participation is evolving and lacks a universally accepted definition. Mars et al. (17) conducted ten individual interviews and two focus groups to identify four domains of social participation: (1) social contacts and social activities; (2) work and informal support; (3) cultural activities and public events; and (4) politics and media. They further delineated three defining characteristics of social participation: social contact, contributing resources to society, and receiving resources from society. Notably, they conceptualized social participation as an inherently positive experience—a distinction from previous definitions, which did not explicitly require a positive valence (17). Zhou et al. (18) synthesized prior studies and proposed five subscales of social participation: social role engagement, activity involvement, use of social resources, interpersonal interaction, and realization of personal values. This framework offered a more specific and simplified conceptualization. Aroogh et al. (19) defined social participation as involvement in social or community activities that involve interpersonal interactions beyond the family. Amini et al. (20), using a grounded theory approach, found that for older adults, staying active is a central concern within the concept of social participation. Despite increasing attention to this construct, there is currently no standardized definition of “social participation” for this population, nor are there efforts to further clarify and standardize it, develop culturally relevant assessment tools, establish a comprehensive support system, and encourage survivors to actively engage in social life to enhance their overall quality of life.

### 2.2 Theoretical framework for social participation

This review is guided by the ICF as the theoretical framework, aiming to refine the conceptualization of social participation, elucidate the mechanisms of its determinants, and provide a theoretical foundation for synthesizing the literature and identifying research gaps.

The ICF framework conceptualizes social participation as a comprehensive functional outcome arising from the interaction among body functions, performance of activities, environmental factors, and personal characteristics (16). The framework emphasizes social participation as a key dimension in evaluating rehabilitation outcomes, thereby providing the theoretical basis for defining the core construct of social participation in young and middle-aged stroke survivors. In the subsequent analysis, the ICF framework guided the selection and critical appraisal of assessment instruments: tools that solely measure a single aspect of body function (e.g., mobility) or that assess generic quality of life were not considered as measures specific to social participation. This approach ensured that the evaluated dimensions remained consistent with the core definition of social participation.

### 2.3 Operational definition of social participation

Based on the ICF framework and considering the unique life stage and role demands of young and middle-aged stroke survivors, this review proposes the following operational definition of social participation in this population: Social participation refers to the active involvement of individuals in activities with interpersonal or social meaning across multiple settings—including family, work, and community. It encompasses three core dimensions: frequency of participation, perceived participation restrictions, and satisfaction with participation. The overarching goals are role renegotiation, social connectedness, and the continuation of personal value.

This review maintains a clear distinction between social participation and community participation. Social participation is conceptualized as the broader construct, encompassing all domains of social life, including family, work, community, and environments. Community participation, in contrast, is treated as a subdomain of social participation, referring specifically to activities occurring within geographic communities (e.g., neighborly interactions, community-based recreational activities). While this review focuses on social participation, studies addressing community participation are cited using their original terminology, with their conceptual placement within the broader ICF framework explicitly clarified in the analysis.

### 2.4 Analysis of the current situation of social participation of young and middle-aged stroke survivors

Social participation is a key element of the quality of life for young and middle-aged stroke survivors, and it is also the ultimate goal of subsequent treatment and nursing (21, 22). However, currently, the level of social participation among young and middle-aged stroke survivors in China is relatively low, and the frequency of social participation has decreased (10), among which the levels of social life and social activities are the lowest (23). To explore the determinants underlying this issue, Zhang et al. (24) conducted a multi-dimensional analysis of social participation among stroke survivors. Their study included 470 Chinese stroke survivors, with a mean age of 62.63 years (range: 37–88 years), including a subset of young and middle-aged individuals (40–60 years). The analysis identified individual-level factors (e.g., anxiety, motivation) and physical environmental factors (e.g., access to medical services) as key determinants of social participation (24). Lin et al. (25) surveyed young and middle-aged stroke survivors one month after discharge and found that stroke severity had the strongest correlation with social participation. Other influencing factors included physical health, psychological state, and social circumstances (15, 26, 27). A 2-year prospective cohort study by Verberne (26) demonstrated that greater dependence in activities of daily living was independently associated with more pronounced restrictions in social participation and poorer trajectories of participation recovery. The study further identified older age and lower educational attainment as significant predictors of lower social engagement levels. The work of Zhuang et al. (27) on young and middle-aged stroke survivors revealed that milder disability, enhanced neurological recovery, and greater ADL independence were strong predictors of better social participation outcomes. Drawing on the ICF framework, Della Vecchia et al. (15) demonstrated that individual coping strategies were significantly associated with social participation levels. Survivors who employed positive thinking as a coping mechanism exhibited higher levels of social participation, whereas those who primarily relied on seeking social support as their main strategy paradoxically showed lower participation. Furthermore, greater satisfaction with one’s living environment was correlated with higher social participation, and perceived satisfaction with social relationships also emerged as a significant determinant of participation outcomes (15). Cross-sectional studies focusing on young and middle-aged stroke survivors have consistently documented low social participation levels, as shown in a survey of 325 survivors by Wen et al. (23) and the work of Wei Juan et al. (28).

## 3 Social participation assessment tool for young and middle-aged stroke survivors

To date, no assessment tools have been specifically developed to evaluate social participation in young and middle-aged stroke survivors (aged 40–60 years); existing instruments are generally designed for the broader adult stroke population (**Table 1**). Accordingly, the tools selected for this review were required to meet the following three criteria: (1) widespread use in both international and domestic research on social participation, community integration, or social reintegration among stroke survivors; (2) established reliability and validity, including validation of Chinese versions, to ensure measurement accuracy and consistency; and (3) design specifically for adult stroke survivors (with demonstrated applicability to younger and middle-aged subgroups) or capacity to indirectly reflect social participation potential by assessing foundational functions essential for social engagement, such as mobility. Where relevant, the specific applicability of each instrument to young and middle-aged survivors will be clarified in the subsequent descriptions to maintain alignment with the core focus of this review.

**Table 1:** Comparison of common assessment tools for social participation in young and middle-aged stroke survivors.

Tool (acronym)	Developer (Year)/chinese version (Year)	Core construct/subscales	Items	Reliability (Cronbach’s α/test-retest)	Key advantages	Limitations/considerations for young and middle-aged stroke survivors
Impact on Participation and Autonomy Questionnaire (IPA)	Cardol et al. (29); He et al. (30)	Indoor autonomy, Outdoor autonomy, Family role, Social life.	25 items; Likert 5-point scale; Total score 0-100. Higher score = lower participation.	0.937 (Chinese version)	Comprehensively assesses perceived autonomy and participation restriction across key life domains. The “autonomy” focus aligns with rehabilitation goals for independence.	The “family role” items may reflect traditional family structures, potentially under-representing modern or diverse family and occupational roles critical to young and middle-aged adults. The inverse scoring can be counterintuitive.
Utrecht Scale for Evaluation of Rehabilitation-Participation (USER-P)	Post et al. (31); Zhang (32)	Frequency, Restrictions, Satisfaction (three subscales).	32 items; Each subscale scored 0-100. Higher score = higher frequency/satisfaction, fewer restrictions.	Frequency: 0.68; Restriction: 0.92; Satisfaction: 0.89 (Chinese version)	Multi-dimensional design uniquely captures objective behavior (frequency), capacity (restriction), and subjective experience (satisfaction).	The frequency subscale’s α (0.68) is slightly below the conventional threshold (0.70), indicating potential instability in this population. Community activity items may not fully capture workplace or social participation relevant to younger adults.
Rivermead Mobility Index (RMI)	Collen et al. (33)	Basic to complex mobility (e.g., turning in bed, walking outdoors).	Original: 15 items, dichotomous scoring (0/1). Modified: 8 items, 6-point scale. Higher score = better mobility.	Modified version: 0.93 (34)	Brief, performance-based, and highly reliable. Mobility is a fundamental prerequisite for community-based social participation.	Not a direct measure of social participation. It assesses a single, albeit critical, component (mobility). Must be used in conjunction with participation-specific tools.
Community Integration Questionnaire (CIQ)	Willer et al. (35)	Home Integration, Social Integration, Productive Activities.	15 items; Total score 0-29. Higher score = better community integration.	Test-retest: 0.91; Subscales: 0.83-0.93	Strong reliability, wide use in brain injury populations. The “productive activities” dimension directly addresses work/school return.	The “productive activities” dimension may have a ceiling effect for unemployed survivors or those unable to return to work, a significant concern for young and middle-aged survivors.
Community Integration Measure (CIM)	McColl et al. (37)	Perceived integration, involvement, and sense of belonging within the community.	10 items; Likert 5-point scale; Score 10-50. Higher score = higher integration.	0.87 (Original); 0.84 (Stroke survivors, Chinese version)	Focuses on the subjective experience of belonging, a key psychological aspect of participation. Short and easy to administer.	May not assess the frequency or diversity of activities. The concept of “community” may be interpreted differently across cultures and urban/rural settings in China.
Reintegration to Normal Living Index (RNLI)	Wood-Dauphinee et al. (39)	Daily Functioning, Self-Perception.	11 items; Typically scored 0-100. Higher score = better reintegration.	0.92 (Stroke survivors, Chinese version)	Excellent reliability (α=0.92 in Chinese stroke sample), sensitive to functional change; dual-dimension design (Daily Functioning + Self-Perception) captures both objective function and subjective perception, reflecting patient-centered goals. Flexible administration (interview/mail), Chinese version validated; intuitive VAS scoring easy for patients.	Items are broad (e.g., “I participate in social activities”). May lack sensitivity to detect subtle changes or specific barriers (e.g., workplace accommodations, online socializing).
Stroke Impact Scale (SIS)	Duncan et al. (41); Qi et al. (Chinese) (42)	8 domains including Strength, Memory, Emotion, ADL/IADL, Mobility, Hand Function, Communication, Participation.	59 items; Each domain scored 0-100. Higher score = better function/QoL.	Domain-specific values reported, generally good.	A comprehensive stroke-specific QoL measure. The Participation domain (8 items) is embedded within a full health profile, useful for holistic assessment.	Lengthy. The Participation domain is not a dedicated measure and does not directly capture social participation compared with specialized tools. It is not designed for standalone use and provides less detail. Its generic items may not target key concerns of young and middle-aged adults.

### 3.1 Impact on Participation and Autonomy Questionnaire

The Impact on Participation and Autonomy Questionnaire (IPA) was developed by Cardol et al. (29) in the Netherlands in 1999, localized into Chinese and revised by Chinese scholars He et al. (30) in 2013, and was mainly used to measure survivors’ social perception and social participation. The IPA consists of 4 subscales, namely indoor autonomy participation (e.g., moving around at home, performing personal care), outdoor autonomy participation (e.g., visiting relatives, traveling for vacation), family role and social life, totaling 25 items. The IPA is scored on a 5-point Likert scale, with a total score of 100 points. Higher scores indicate lower levels of participation and autonomy. The original Dutch version of the IPA demonstrated good internal consistency, with Cronbach’s α coefficients ranging from 0.84 to 0.87 for its four subscales (29). The Chinese version (IPA-I) also showed excellent reliability, with an overall Cronbach’s α of 0.937, and subscale alphas ranging from 0.667 to 0.965 (30).

### 3.2 Utrecht Scale for Evaluation of Rehabilitation-Participation

The Utrecht Scale for Evaluation of Rehabilitation-Participation (USER-P) was developed by Post et al. (31) in the Netherlands in 2011 and was localized into Chinese and validated for reliability by Chinese scholars Zhang et al. (32) in 2016.The USER-P is widely used to assess social participation in stroke survivors. As a comprehensive instrument for assessing rehabilitation participation, the USER-P covers multiple life domains, including paid work, unpaid voluntary work, education/training, household activities, leisure pursuits, and social interactions. The scale comprises 32 items distributed across three subscales: the Frequency scale (11 items), the Restrictions scale (11 items), and the Satisfaction scale (10 items). Scores for each subscale are transformed to a 0–100 range, with higher scores indicating greater participation frequency, fewer restrictions, and higher satisfaction, respectively. In particular, items pertaining to social interactions (e.g., visiting others, receiving visitors, maintaining contact), outings (e.g., dining out, short trips), and leisure participation directly capture core dimensions of social engagement, rendering the USER-P particularly suitable for assessing social participation in stroke survivors. The Chinese version of the USER-P demonstrated acceptable to excellent internal consistency, with Cronbach’s α coefficients of 0.681, 0.920, and 0.885 for the Frequency, Restrictions, and Satisfaction subscales, respectively.

### 3.3 Rivermead Mobility Index

The Rivermead Mobility Index (RMI) was developed by British scholars Collen et al. (33) in 1991 at the Rivermead Rehabilitation Center. It is mainly used to evaluate the mobility of survivors with stroke and other neurological diseases. The RMI consists of 14 items and one direct observation, covering the assessment of dynamic mobility from basic activities (such as turning over in bed and sitting up) to complex functions (such as going up and down stairs and walking outdoors). Each task is scored as “0 points (unable to complete)” or “1 point (completed independently)” according to the patient’s performance, with a total of 15 points. The higher the score, the better the patient’s mobility function level (33). A subsequent abbreviated version was developed by Lennon et al. (34), who reduced the number of items from 15 to 8 and replaced the dichotomous scoring with a 6-point scale. This shortened version maintains a high correlation with the original RMI while reducing administration time, and demonstrates excellent internal consistency, with a Cronbach’s α of 0.93 (34). Although primarily a measure of mobility, the RMI was included in this review for its clear conceptual alignment with the ICF framework. Within the ICF model, mobility represents a component of body function and activity performance, which serves as the most basic, necessary physical prerequisite for social participation. As a widely used, stroke-specific instrument for measuring physical mobility capacity, the RMI directly reflects the foundational physical function that enables social participation. For young and middle-aged stroke survivors—who face urgent demands of returning to work and resuming family roles—independent mobility is particularly critical, making the RMI a targeted and valid measure for this population.

### 3.4 Community Integration Questionnaire

The Community Integration Questionnaire (CIQ) was developed by Willer et al. (35) in New York in 1993. It is designed to assess the degree of community integration achieved by individuals with brain injuries in family, community, and social activities (e.g., planning social gatherings, going out shopping, engaging in leisure and recreational activities, and participating in social activities with companions). The CIQ consists of 3 subscales, namely family integration, social integration, and productive activities, with a total of 15 items. The total score ranges from 0 to 29 points, and the higher the score, the better the level of community integration. The overall test-retest reliability of the questionnaire is 0.91, with subscale test-retest reliabilities ranging from 0.83 to 0.93. Its reliability and validity have been widely verified in stroke and traumatic brain injury survivors (35, 36).

### 3.5 Community Integration Measure

The Community Integration Measure (CIM) was developed by McColl et al. (37) in 2001 based on qualitative research with individuals with acquired brain injury. It assesses the extent to which individuals participate in community activities, feel connected to their community, and experience a sense of belonging. This 10-item self-report scale uses a 5-point Likert response format, with total scores ranging from 10 to 50. Higher scores reflect greater community integration. The CIM has demonstrated good reliability and validity in stroke populations, with Cronbach’s α coefficients of 0.87 in the original validation (36) and 0.84 in subsequent stroke-specific studies (38). Although community participation and social participation are distinct constructs, the CIM was included because it directly assesses core social participation components (e.g., social relationships, community leisure engagement, interpersonal interactions) and has significant positive correlations with validated social participation (CIQ) and social support (ISEL) measures (37). For young and middle-aged stroke survivors, the CIM’s focus on community-based social participation performance aligns with their key goal of resuming social life in daily community scenarios, providing valuable supplementary assessment information.

### 3.6 Reintegration to Normal Living Index

The Reintegration to Normal Living Index (RNLI) was developed by Wood-Dauphinee et al. (39) in 1988. It is mainly used to measure the degree to which individuals with traumatic or neurological disorders are reintegrated into normal social activities. This scale is a self-assessment tool, divided into two subscales: daily function dimension and self-perception dimension, with a total of 11 items. Higher total scores indicate greater reintegration into normal life. The RNLI has demonstrated strong psychometric properties in stroke populations. Cronbach’s α coefficients range from 0.87 in the original validation (39) to 0.92 in stroke-specific studies, with test-retest reliability of 0.87 (40).

### 3.7 Stroke Impact Scale

The SIS was developed by Duncan et al. (41) in 1999 and localized into Chinese by Qi et al. (42) in 2007. It is widely used to assess health-related quality of life in stroke survivors. The original SIS Version 2.0 consists of 8 domains with a total of 64 items, namely strength, memory and thinking, communication, ADL/IADL (Activities of Daily Living/Instrumental Activities of Daily Living), mobility, hand function, emotion, and participation. The Chinese version was revised to 59 items on the basis of the original scale. All items are rated on a 5-point Likert scale, with higher scores indicating better health-related quality of life in stroke survivors. Although the SIS was developed as a comprehensive quality-of-life instrument rather than a dedicated measure of social participation, its Participation domain directly assesses the impact of stroke on social participation areas—including work, social activities, and family roles. This domain has demonstrated strong positive correlations with the Social Functioning subscale of the 36-Item Short Form Survey (SF-36), a well-validated measure of social functioning. For young and middle-aged stroke survivors, for whom returning to work and resuming social roles are paramount, the SIS provides a comprehensive reflection of participation restrictions and recovery trajectories. Accordingly, it was included in this review.

### 3.8 Synthesis and critical appraisal of assessment tools

Based on the operational definition of social participation established in this review, the assessment instruments described above differ substantially in their scope and focus. (1) For comprehensive, multidimensional assessment of social participation, the USER-P is recommended as the preferred instrument, as its three-subscale structure enables simultaneous evaluation of participation frequency, restrictions, and satisfaction. (2) The IPA offers unique value in assessing perceived autonomy—a dimension closely linked to psychological adaptation during rehabilitation. (3) For rapid screening or large-scale surveys, brief instruments such as the CIM and the RNLI offer greater feasibility, though they provide limited detail regarding specific activities and contextualized participation. When considered from the perspective of young and middle-aged stroke survivors, however, existing instruments exhibit notable limitations. None of these measures were specifically designed for this population, and key life domains central to young and middle-aged adults—including occupational reengagement, family caregiving roles, social interaction, and participation in complex social networks—are either absent, inadequately addressed or only superficially covered. Furthermore, the psychometric properties of these tools (e.g., construct validity, measurement invariance) have rarely been validated specifically in this age group, limiting their applicability and interpretability in young and middle-aged stroke populations.

## 4 Discussion

### 4.1 Factors affecting the level of social participation of young and middle-aged stroke survivors

Based on the ICF framework presented earlier, the factors influencing the social participation of young and middle-aged stroke survivors are systematically classified and analyzed into four dimensions: personal characteristics, body functions, performance of activities, and environmental factors.

#### 4.1.1 Personal characteristics

##### 4.1.1.1 Influence of literacy

Cultural literacy is also one of the key demographic factors affecting the social participation of young and middle-aged stroke survivors (43, 44). This study focuses specifically on individuals aged 40–60 years, a population in which education level plays a significant positive role in shaping post-stroke social engagement. Higher educational attainment enhances cognitive reserve by activating compensatory neural networks, thereby strengthening the capacity to process complex information. This, in turn, supports the preservation of cognitive function following stroke and facilitates sustained social participation (43). Moreover, individuals with higher education are better equipped to access, evaluate, and apply rehabilitation-related knowledge. They are more likely to engage with health platforms—such as mobile health applications and online rehabilitation communities—to optimize self-management, which promotes active involvement in diverse social roles, including return to work, family caregiving, and participation in social networks (45). In contrast, survivors with lower educational attainment often exhibit limited health literacy and reduced health-related cognition, which may lead to maladaptive beliefs about medication and negatively affect adherence to secondary prevention therapies (46). Within this population, post-stroke sequelae frequently compromise engagement in parenting and household responsibilities, resulting in a marked decline in participation in family and social roles. Higher educational attainment serves as a critical foundation for vocational adaptation and the successful resumption of these roles. Specifically, stroke survivors with higher levels of education are more likely to achieve stable and sustained return to work, thereby enhancing their overall level of social participation (47–49).

##### 4.1.1.2 Psychological and emotional factors

Psychological and emotional factors play a critical role in shaping social participation among young and middle-aged stroke survivors. Unlike older adults, this population—often serving as primary breadwinners and core members of the workforce—frequently experiences a role identity crisis following stroke-induced physical impairments, which can trigger a cascade of negative emotional responses, including anxiety, depression, helplessness, and disease-related shame. Notably, the prevalence of such emotional disturbances is significantly higher in younger stroke cohorts compared to their older counterparts (50). Even among survivors who achieve favorable physical recovery through rehabilitation, the psychological burden imposed by negative emotions can substantially diminish rehabilitation motivation and willingness to engage socially, sometimes precipitating social avoidance behaviors. This, in turn, exacerbates social isolation and constitutes a major psychological barrier to resuming family roles, returning to work, and reintegrating into society (50). Evidence confirms that psychological motivation is a core predictor of social participation after stroke. The erosion of motivation resulting from negative emotional states can directly foster resistance to social engagement and occupational reintegration, with its detrimental impact on participation occurring independently of the degree of physical impairment (51). Furthermore, discrepancies in illness perceptions between survivors and their caregivers may exacerbate psychological distress, reduce engagement in daily activities, and indirectly impede the recovery of social participation capacity (52). Therefore, clinical management of young and middle-aged stroke survivors should prioritize psychological and emotional regulation. Tailored psychological interventions—such as hope theory-based empowerment nursing and nurse-led peer support programs—have proven effective in alleviating psychological distress, enhancing hope and self-efficacy, reducing disease-related shame, and improving both the frequency and satisfaction of social participation in this population (50, 53).

#### 4.1.2 Body functions

##### 4.1.2.1 Neurological function deficit

Studies have shown that neurological function deficit can affect the social participation of young and middle-aged stroke survivors, and the more severe the neurological function deficit, the poorer the social participation of the survivors (54). Neurological function deficits can lead to problems such as hemiplegia, hemianopia, limb movement disorders, numbness, and cognitive impairment in survivors, which limit the survivors’ ability to perform activities of daily living, making it difficult for them to return to work normally and reducing their interpersonal communication and social participation abilities (27). In addition, post-stroke cognitive impairment is one of the severe complications of stroke (55). Damage to the cognitive areas of the brain leads to a decline in memory, attention, and thinking abilities. This cognitive impairment is significantly associated with reduced ability to participate in complex social activities, work, and interpersonal relationships, making it difficult for survivors to adapt to social environments (56, 57). Disability severity serves as a quantified composite measure of post-stroke neurological impairment and represents a downstream determinant of reduced social participation in young and middle-aged stroke survivors (58). Evidence indicates that young and middle-aged survivors with severe post-stroke disability are particularly susceptible to declines in both motor and cognitive function, with such declines being frequently accompanied by more pronounced impairments in social participation (59, 60). Building upon the physical dysfunction resulting from neurological deficits, severe disability results in persistent motor and speech limitations. These persistent functional impairments, in turn, predispose survivors to negative emotional states such as anxiety and low self-esteem (60, 61). At the same time, stroke survivors frequently encounter stigmatizing attitudes and discriminatory experiences during their rehabilitation phase. Such experiences can contribute to a state of self-deprecation, ultimately manifesting as a shrinking social network and reduction in social engagement (62). Therefore, in clinical practice, healthcare professionals should implement targeted interventions aimed at actively preventing and managing post-stroke neurological deficits. Early rehabilitation interventions to mitigate the resulting disability severity not only contribute to improving the quality of life of young and middle-aged stroke survivors but also establish the physical foundation necessary for restoring and enhancing their social participation.

##### 4.1.2.2 Stroke recurrence

Lin et al. (63) conducted interviews with stroke survivors and found that those with a higher number of recurrences experienced a marked decline in self-care ability; moreover, the greater the frequency of recurrence, the more severe the deterioration in self-care, eventually leading to complete dependency in some cases. This is likely attributable to the secondary brain injury caused by recurrent stroke events, which exacerbates impairments in body structure and function. Specifically, on one hand, lesion expansion or the development of new lesions due to recurrence can further disrupt motor pathways, resulting in muscle weakness, balance dysfunction, and other motor deficits—domains that are fundamental to survivors’ engagement in daily social interactions and occupational activities; On the other hand, recurrence-related neurological damage can also intensify cognitive decline (e.g., memory and attention) and executive dysfunction (e.g., planning and decision-making), directly compromising survivors’ ability to comprehend and execute social tasks, thereby reducing their capacity for social interaction. The compounding effect of these multidimensional functional deficits leads recurrent stroke survivors to vividly perceive a significant impairment in their ability to participate in society (64). For young and middle-aged stroke survivors—a distinct subgroup—the negative impact of recurrence on social participation is particularly pronounced. As primary breadwinners and core members of the workforce, these individuals are typically in the ascendant phase of their careers and hold substantially higher expectations for returning to work, fulfilling family responsibilities, and maintaining social roles compared to older survivors (65). However, the persistent functional impairments resulting from stroke recurrence often directly derail their professional trajectories: survivors are frequently unable to perform their previous jobs due to motor limitations or reduced cognitive efficiency, and even those who retain basic work capabilities often struggle to meet the complex demands of occupational tasks and social interactions (64). Simultaneously, recurrence-induced declines in self-care capacity undermine their ability to shoulder family responsibilities such as childcare and eldercare, forcing a reconfiguration of their familial roles. This disruption engenders a profound sense of role identity crisis and hopelessness about life (65), ultimately culminating in a marked reduction in their level of social participation.

#### 4.1.3 Performance of activities

##### 4.1.3.1 Ability to perform activities of daily living

The ability to perform activities of daily living (ADLs) has an impact on the social participation of young and middle-aged stroke survivors; the better the ADLs, the higher the social participation of the survivors (23). Reduced ability to perform activities of daily living affects the patient’s ability to perform social activities and reduces the patient’s social functioning. survivors with physical dysfunction have to rely on others in their daily lives, and in order not to add unnecessary trouble to others, the patient takes the initiative to reduce social participation (66). Goh et al. (67) demonstrated that social participation is a key determinant of quality of life among urban elderly stroke survivors (aged ≥60 years) in developing countries. Although their study focused on an older cohort, this core finding is consistent with the systematic review evidence from Zhou et al. (68), which included stroke survivors across age groups and confirmed the robust association between social participation and quality of life—an association also observed in the present study of young and middle-aged stroke survivors. Therefore, in clinical practice, healthcare professionals should prioritize survivors’ capacity to perform activities of daily living. By providing early rehabilitation therapy and training, they can help minimize disability, enhance self-care and daily functioning, and ultimately promote greater social participation. (69).

##### 4.1.3.2 Autonomy

Autonomy, defined as the core capacity to independently perform daily activities and make personal decisions and choices, is an important determinant of social participation in young and middle-aged stroke survivors (70). For young and middle-aged adults—who often serve as primary breadwinners and core members of the workforce—the restoration of autonomy is not merely a key marker of physical recovery, but also a critical prerequisite for resuming family roles, re-engaging in employment, and reintegrating into society (25). Evidence confirms that encouraging survivors to maintain indoor autonomy in daily activities and preserve basic social connections during the acute rehabilitation phase significantly improves their autonomy and social participation levels one year after stroke onset (58); Early experiences of autonomy serve to enhance survivors’ self-efficacy, which, in turn, motivates them to engage more actively in rehabilitation training and social interaction (51, 70). However, current evidence indicates that perceived autonomy is often low among stroke survivors (71), and the loss of autonomous capacity—particularly the ability to function independently outdoors—can engender feelings of helplessness. This, in turn, may precipitate social withdrawal, posing a significant barrier to maintaining social participation (72). Therefore, clinical practice should encourage survivors to maintain autonomous activities as early as possible, provided that safety is not compromised, in order to consolidate self-efficacy. Concurrently, close attention should be paid to feelings of helplessness arising from lost autonomy, and targeted interventions should be implemented to prevent the onset of social withdrawal.

#### 4.1.4 Environmental factors

##### 4.1.4.1 Economic status

Numerous studies have demonstrated that socioeconomic status is a factor influencing the social engagement of young and middle-aged stroke survivors (23, 27, 66, 73). Financial disparities can impact their participation in society on several levels. Survivors with higher incomes or better financial stability are generally more likely to engage in social activities, access rehabilitation services, and reintegrate into the community more effectively (23). This may be because survivors with better economic conditions are not restricted by economic factors in terms of rehabilitation resources and can easily obtain guidance on the latest cutting-edge rehabilitation knowledge or new treatment technologies. In contrast, low-income survivors are restricted by economic factors and find it difficult to afford long-term rehabilitation costs, which may lead to treatment interruption and poor functional recovery (73). On the other hand, economic pressure can intensify psychological stress. Survivors often feel self-blame and guilt because the disease brings an economic burden to their families (66). Facing high medical expenses and uncertain economic situations, they are prone to develop anxiety and depression, and may even reduce their compliance with rehabilitation treatment (74, 75). Such negative psychological states and poor rehabilitation adherence will further impede physical function recovery, thereby ultimately compromising the social participation of young and middle-aged stroke survivors (70, 74). In addition, the medical security system (a multi-level medical security system in China with basic medical insurance as the main body, supplemented by commercial health insurance, medical assistance and other forms) can mitigate these economic constraints. Zhuang et al. (27) reported that young and middle-aged stroke survivors with commercial insurance exhibited higher levels of social participation.

### 4.2 Limitations

This review synthesizes existing research on social participation in young and middle-aged stroke survivors, yet several limitations must be acknowledged. (1) Currently, there is no standardized conceptual definition of social participation specifically for this population, nor are there specialized assessment instruments. (2) Most included studies employed cross-sectional designs, resulting in insufficient longitudinal data on the dynamic trajectories and underlying mechanisms of social participation during neurorehabilitation. Consequently, this review cannot systematically delineate causal relationships between influencing factors and social participation levels. (3) Intervention studies targeting social participation in this population are limited, with narrow research scopes and small sample sizes, precluding in-depth analysis of intervention effects. Future research should prioritize tailored interventions (e.g., vocational reintegration, resumption of family caregiving roles, reduction of disease-related stigma) to enhance their social participation. (4) While findings (e.g., impacts of economic status, educational attainment, and psychological factors) are consistent with international literature, suggesting cross-cultural relevance, the specific manifestations of social participation, pathways of influencing factors, and moderating effects of macro-level factors (e.g., healthcare policies, cultural norms) may vary across regions. Thus, the applicability of these findings to other cultural and healthcare contexts warrants further validation through cross-national comparative studies. (5) As a narrative review, this study lacked systematic literature screening, quality assessment, and quantitative synthesis, leading to a limited evidence level of conclusions. Future verification through systematic reviews or meta-analyses is needed.

## 5 Conclusion

Stroke, as the third leading cause of death globally, shows a gradually rising prevalence trend among young and middle-aged people in China. Its high disability rate leads to a decline in the social participation level of survivors, severely affecting their quality of life. Social participation plays a crucial role in the physical and mental rehabilitation of survivors by promoting neural remodeling and improving cognitive function. However, existing studies indicate that the social participation level of young and middle-aged stroke survivors is generally low. Although current assessment tools are widely used, there is a lack of specialized scales designed based on the ICF framework specifically for this group. In addition, there are few intervention studies on the social participation of young and middle-aged stroke survivors. The research scope is limited, the research population is relatively narrow, and the methods and effects of the interventions need further verification. Therefore, future studies could deeply explore the influencing factors of social participation among young and middle-aged stroke survivors from the perspective of the ICF framework’s dimensions (personal characteristics, body functions, performance of activities, and environmental factors) to predict the level of social participation, and develop culturally appropriate assessment tools across different regions. It is necessary to establish and improve a comprehensive support and intervention system covering all dimensions of social participation (e.g., social interaction, occupational reintegration, family role fulfillment, and community participation) for young and middle-aged stroke survivors, so as to facilitate their role renegotiation, social connectedness and the continuity of personal value, thereby enhancing their social participation, improving their quality of life, and optimizing health outcomes.

## References

[1] Global, regional, and national burden of stroke and its risk factors, 1990-2021: a systematic analysis for the Global Burden of Disease Study 2021. Lancet Neurol. (2024) 23:973–1003. doi: 10.1016/s1474-4422(24)00369-7. PMID: 39304265 PMC12254192

[2] R.o.S.C.i.C.W. Group . Brief report on stroke center in China, 2022. Chin J Cerebrovascular Dis. (2024) 21:565–76.

[3] Guo X Xue Q Zhao J Yang Y Yu Y Liu D . Clinical diagnostic and therapeutic guidelines of stroke neurorestoration (2020 China version). J Neurorestoratology. (2020) 8:241–51. doi: 10.26599/jnr.2020.9040026. PMID: 41311685

[4] Passarelli JP Nimjee SM Townsend KL . Stroke and neurogenesis: bridging clinical observations to new mechanistic insights from animal models. Transl Stroke Res. (2024) 15:53–68. doi: 10.1007/s12975-022-01109-1. PMID: 36462099

[5] Olver J Yang S Fedele B Ni J Frayne J Shen G . Post stroke outcome: global insight into persisting sequelae using the post stroke checklist. J Stroke Cerebrovasc Dis. (2021) 30:105612. doi: 10.1016/j.jstrokecerebrovasdis.2021.105612. PMID: 33493876

[6] Wu Y Li Z Feng K Cheng Y Wang Y Yin S . Prognostic factors of prolonged disorder of consciousness after stroke: a single center retrospective study. J Neurorestoratology. (2023) 11(1). doi: 10.1016/j.jnrt.2022.100032. PMID: 41916819

[7] R.o.S.P.a.T.i.C.W. Group . Brief report on stroke prevention and treatment in China, 2021. Chin J Cerebrovascular Dis. (2023) 20:783–93.

[8] Ning X Sun J Jiang R Lu H Bai L Shi M . Increased stroke burdens among the low-income young and middle aged in rural China. Stroke. (2017) 48:77–83. doi: 10.1161/strokeaha.116.014897. PMID: 27924051

[9] Qian Z Ruijun J Meng Z Wenjuan W Jingjing L Na L . Chinese stroke association guidelines for clinical management of cerebrovascular diseases (Second edition)(Excerpt)--chapter five clinical management of intracerebral hemorrhage. Chin J Stroke. (2023) 18:1014–23.

[10] Yi L Lifeng Z Genqun W . Study on social participation and its influencing factors of young and middle-aged stroke patients. Chin Gen Pract Nurs. (2024) 22:1905–10.

[11] Fang X Wang Y Wang X Wang R Yuqing M Luo S . The impact of activities of daily living on cognitive function in elderly individuals with chronic comorbidities: the chain mediating effects of social participation and depression. Modern Prev Med. (2024) 51:3576–82. doi: 10.20043/j.cnki.MPM.202405325

[12] Qingling Z Sisi Y . Research progress on social participation and its influencing factors of the elderly in community. Chin Nurs Manage. (2018) 18:1293–6.

[13] Yiting L Jianqian C Xueyu W . The prevalence of comorbid chronic medical conditions and influencing factors in elderly residents in Nanjing. Chin Prev Med. (2022) 23:646–51. doi: 10.16506/j.1009-6639.2022.09.002

[14] Obembe AO Eng JJ . Rehabilitation interventions for improving social participation after stroke: a systematic review and meta-analysis. Int J Stroke. (2015) 10:94. doi: 10.1177/1545968315597072. PMID: 26223681 PMC4868548

[15] Della Vecchia C Préau M Haesebaert J Viprey M Rode G Termoz A . Factors associated with post-stroke social participation: a quantitative study based on the ICF framework. Ann Phys Rehabil Med. (2023) 66:101686. doi: 10.1016/j.rehab.2022.101686. PMID: 35779831

[16] Xuan Z . Difficult regression: the change and development process of social participation in stroke patients. In: Chinese People’s Liberation Army Naval Medical University. Shanghai, China: Naval Medical University of the Chinese People’s Liberation Army (2019).

[17] Mars GM Kempen GI Mesters I Proot IM Van Eijk JT . Characteristics of social participation as defined by older adults with a chronic physical illness. Disabil Rehabil. (2008) 30:1298–308. doi: 10.1080/09638280701623554. PMID: 17882727

[18] Xuan Z Xiuhua T Lanshu Z . Research progress on the concept of social participation. Chin J Rehabil Med. (2018) 33:475–8.

[19] Dehi Aroogh M Mohammadi Shahboulaghi F . Social participation of older adults: a concept analysis. Int J Community Based Nurs Midwifery. (2020) 8:55–72. doi: 10.30476/ijcbnm.2019.82222.1055. PMID: 32039280 PMC6969951

[20] Amini R Shahboulaghi FM Tabrizi KN Forouzan AS . Social participation among Iranian community-dwelling older adults: a grounded theory study. J Family Med Prim Care. (2022) 11:2311–9. doi: 10.4103/jfmpc.jfmpc_1775_21. PMID: 36119239 PMC9480767

[21] de Diego-Alonso C Bellosta-López P Blasco-Abadía J Buesa-Estéllez A Roldán-Pérez P Medina-Rincón A . The relationship between levels of physical activity and participation in everyday life in stroke survivors: a systematic review and meta-analysis. Disabil Health J. (2024) 17:101640. doi: 10.1016/j.dhjo.2024.101640. PMID: 38777677

[22] Yirong X Mohamad NA Said FM Tan BG . A review of the social participation of stroke survivors. Int J Emerging Issues Soc Science Arts Humanities. (2024) 3(1). doi: 10.60072/ijeissah.2024.v3i01.007

[23] Xiaofen W Fanshan H Yan L Na O Jiangping C Xiaoping H . Study on the level of social participation and its influencing factors in young and middle-aged patients with ischemic stroke. J Med Theory Pract. (2024) 37:2482–5. doi: 10.19381/j.issn.1001-7585.2024.14.052

[24] Zhang H Liu W Sun Y Ma L Zhang D Wu XV . Multi-dimensional factors associated with adequate social participation among stroke survivors based on the social ecological model: a cross-sectional study on the gender and living place differences. Geriatr Nurs. (2024) 60:654–63. doi: 10.1016/j.gerinurse.2024.10.042. PMID: 39515148

[25] Xiao L Gao Y Keng K Zhang L . Perceived participation and its determinants among young and middle-aged stroke survivors following acute care one month after discharge. Disabil Rehabil. (2021) 43:648–56. doi: 10.1080/09638288.2019.1636314. PMID: 31437066

[26] Verberne DPJ Post MWM Köhler S Carey LM Visser-Meily JMA van Heugten CM . Course of social participation in the first 2 years after stroke and its associations with demographic and stroke-related factors. Neurorehabil Neural Repair. (2018) 32:821–33. doi: 10.1177/1545968318796341. PMID: 30178696 PMC6146317

[27] Yanjun Z Runluo S Zixiu Z Dandan S Jing K . Study on the present status and influencing factors of social participation level for young and middle-aged stroke patient. J Nurs Rehabil. (2022) 21:5–9.

[28] Juan W Ting W Qiaowei L Cuiyun Z Zhenzhen Z . Status quo and influencing factors on social participation feeling of stroke patients. Chin Nurs Res. (2023) 37:321–6.

[29] Cardol M de Haan RJ van den Bos GA de Jong BA de Groot IJ . The development of a handicap assessment questionnaire: the Impact on Participation and Autonomy (IPA). Clin Rehabil. (1999) 13:411–9. doi: 10.1191/026921599668601325. PMID: 10498348

[30] Yanan H Xia W Hong L Lanshu Z . The validity and reliability of the Chinese version of the lmpact on Participation and Autonomy Questionnaire among stroke patients. Chin Nurs Manage. (2013) 13:22–4.

[31] Post MW van der Zee CH Hennink J Schafrat CG Visser-Meily JM van Berlekom SB . Validity of the utrecht scale for evaluation of rehabilitation-participation. Disabil Rehabil. (2012) 34:478–85. doi: 10.3109/09638288.2011.608148. PMID: 21978031

[32] Zhiying Z . The Chinesization and reliability and validity verification of the Utrecht Rehabilitation-Participation Assessment Scale. In: suzhou university. Suzhou, China: Soochow University (2016).

[33] Collen FM Wade DT Robb GF Bradshaw CM . The Rivermead Mobility Index: a further development of the Rivermead Motor Assessment. Int Disabil Stud. (1991) 13:50–4. doi: 10.3109/03790799109166684. PMID: 1836787

[34] Lennon S Johnson L . The modified rivermead mobility index: validity and reliability. Disabil Rehabil. (2000) 22:833–9. doi: 10.1080/09638280050207884. PMID: 11197520

[35] Willer B Ottenbacher KJ Coad ML . The community integration questionnaire. A comparative examination. Am J Phys Med Rehabil. (1994) 73:103–11. doi: 10.1097/00002060-199404000-00006. PMID: 8148099

[36] Shuaiyou W Dingding L Chenjun L Xueting S Yage S Hongru W . Research progress of community integration assessment tools for stroke patients. Chin Nurs Res. (2024) 38:4041–6.

[37] McColl MA Davies D Carlson P Johnston J Minnes P . The community integration measure: development and preliminary validation. Arch Phys Med Rehabil. (2001) 82:429–34. doi: 10.1053/apmr.2001.22195. PMID: 11295000

[38] Liu TW Ng SS Ng GY . Translation and initial validation of the Chinese (Cantonese) version of community integration measure for use in patients with chronic stroke. BioMed Res Int. (2014) 2014:623836. doi: 10.1155/2014/623836. PMID: 24995317 PMC4065661

[39] Wood-Dauphinee SL Opzoomer MA Williams JI Marchand B Spitzer WO . Assessment of global function: the Reintegration to Normal Living Index. Arch Phys Med Rehabil. (1988) 69:583–90. doi: 10.1037/t28919-000. PMID: 3408328

[40] Pang MY Lau RW Yeung PK Liao LR Chung RC . Development and validation of the Chinese version of the Reintegration to Normal Living Index for use with stroke patients. J Rehabil Med. (2011) 43:243–50. doi: 10.2340/16501977-0660. PMID: 21305241

[41] Duncan PW Wallace D Lai SM Johnson D Embretson S Laster LJ . The stroke impact scale version 2.0. Evaluation of reliability, validity, and sensitivity to change. Stroke. (1999) 30:2131–40. doi: 10.1161/01.str.30.10.2131. PMID: 10512918

[42] Minghua Q Yuqian T Lingyan W Lizhi W . Translation and psychometric evaluation of the Stroke Impact Scale 3.0 for Proxy in Chinese version. Chin J Tissue Eng Res. (2007), 5920–4.

[43] Oliva G Masina F Hosseinkhani N Montemurro S Arcara G . Cognitive reserve in the recovery and rehabilitation of stroke and traumatic brain injury: a systematic review. Clin Neuropsychologist. (2025) 39:1450–86. doi: 10.1080/13854046.2024.2405226. PMID: 39307973

[44] Cai Y Towne SD Bickel CS . Multi-level factors associated with social participation among stroke survivors: China's Health and Retirement Longitudinal Study (2011-2015). Int J Environ Res Public Health. (2019) 16(24):5121. doi: 10.3390/ijerph16245121. PMID: 31847437 PMC6950688

[45] Vu MTT Ho HQ Lin GH . eHealth interventions of health literacy for stroke survivors: systematic review and meta-analysis. Public Health Nurs. (2025) 42:516–23. doi: 10.1111/phn.13432. PMID: 39344209

[46] Ruksakulpiwat S Benjasirisan C Ding KD Phianhasin L Thorngthip S Ajibade A . Utilizing social determinants of health model to understand barriers to medication adherence in patients with ischemic stroke: a systematic review. Patient Preference Adherence. (2023) 17:2161–74. doi: 10.2147/ppa.S420059. PMID: 37667687 PMC10475305

[47] Westerlind E Persson HC Eriksson M Norrving B Sunnerhagen KS . Return to work after stroke: a Swedish nationwide registry-based study. Acta Neurol Scand. (2020) 141:56–64. doi: 10.1111/ane.13180. PMID: 31659744 PMC6916554

[48] Harris GM Bettger JP . Parenting after stroke: a systematic review. Topics Stroke Rehabil. (2018) 25:384–92. doi: 10.1080/10749357.2018.1452366. PMID: 29607739

[49] Xiaonan W Lingmin Z Li F Jia Z Mingxing Z Ning L . Health-related quality of life in rural stroke patients in a certain area of western China. Chin J Gerontology. (2021) 41:1511–4.

[50] Wu XJ Ke K Liu H Zhan SP Wang L He JF . Social isolation in the young and middle-aged patients with stroke: role of social support, family resilience and hope. Front Psychiatry. (2025) 16:1499186. doi: 10.3389/fpsyt.2025.1499186. PMID: 40012713 PMC11861369

[51] Gingrich N Bosancich J Schmidt J Sakakibara BM . Capability, opportunity, motivation, and social participation after stroke. Top Stroke Rehabil. (2023) 30:423–35. doi: 10.1080/10749357.2022.2070358. PMID: 35510695

[52] Shi Y Howe TH Halpin PF Hu L Wu B . Dyadic analysis of illness perceptions among individuals with stroke and their caregivers: effects on activity engagement in community living. Disabil Rehabil. (2024) 46:3342–54. doi: 10.1080/09638288.2023.2246378. PMID: 37602644

[53] Qianqian S Yongxia M Qingxian L Zhiguang P Juanjuan R Zhenxiang Z . The potential categories of post-traumatic growth and its relationship with rumination and depression in young and middle-aged stroke patients. Chin J Prev Control Chronic Dis. (2024) 32:522–6. doi: 10.16386/j.cjpccd.issn.1004-6194.2024.07.009

[54] Elloker T Rhoda A Arowoiya A Lawal IU . Factors predicting community participation in patients living with stroke, in the Western Cape, South Africa. Disabil Rehabil. (2019) 41:2640–7. doi: 10.1080/09638288.2018.1473509. PMID: 29842817

[55] Wei X Ma Y Wu T Yang Y Yuan Y Qin J . Which cutoff value of the Montreal Cognitive Assessment should be used for post-stroke cognitive impairment? A systematic review and meta-analysis on diagnostic test accuracy. Int J Stroke. (2023) 18:908–16. doi: 10.1177/17474930231178660. PMID: 37190789

[56] El Husseini N Katzan IL Rost NS Blake ML Byun E Pendlebury ST . Cognitive impairment after ischemic and hemorrhagic stroke: A scientific statement from the american heart association/american stroke association. Stroke. (2023) 54:e272–91. doi: 10.1161/str.0000000000000430. PMID: 37125534 PMC12723706

[57] Stolwyk RJ Mihaljcic T Wong DK Chapman JE Rogers JM . Poststroke cognitive impairment negatively impacts activity and participation outcomes: A systematic review and meta-analysis. Stroke. (2021) 52:748–60. doi: 10.1161/strokeaha.120.032215. PMID: 33493048

[58] Törnbom K Hadartz K Sunnerhagen KS . Self-Perceived Participation and Autonomy at 1-Year Post Stroke: A Part of the Stroke Arm Longitudinal Study at the University of Gothenburg (SALGOT Study). J Stroke Cerebrovasc Dis. (2018) 27:1115–22. doi: 10.1016/j.jstrokecerebrovasdis.2017.11.028. PMID: 29284572

[59] Mussa R Ambler G Ozkan H Mitchell J Banerjee A Leff AP . Patient-reported outcomes after stroke in young adults: University College London (UCL) Young Stroke Systematic Evaluation Study (ULYSSES). J Neurol Neurosurg Psychiatry. (2025) 97:3–12. doi: 10.1136/jnnp-2025-336411. PMID: 41072956

[60] Naess H Waje-Andreassen U Thomassen L Nyland H Myhr KM . Health-related quality of life among young adults with ischemic stroke on long-term follow-up. Stroke. (2006) 37:1232–6. doi: 10.1161/01.Str.0000217652.42273.02. PMID: 16601213

[61] Ayerbe L Ayis S Wolfe CD Rudd AG . Natural history, predictors and outcomes of depression after stroke: systematic review and meta-analysis. Br J Psychiatry. (2013) 202:14–21. doi: 10.1192/bjp.bp.111.107664. PMID: 23284148

[62] Juanamasta IG Polsook R Aungsuroch Y Kariasa IM Gunawan J . Being otherness: A phenomenological journey of stroke survivors' Stigma. J Prim Care Community Health. (2025) 16:21501319251339375. doi: 10.1177/21501319251339375. PMID: 41170591 PMC12579181

[63] Beilei L Yunfei G Zhenxiang Z Yongxia M Junli S Xihong X . A qualitative study on disease experience and recurrence risk perception of stroke survivors. Chin J Nurs. (2021) 56:80–5.

[64] Ribeiro de Souza F Sales M Rabelo Laporte L Melo A Nmanoel da Silva Ribeiro N . Body structure/function impairments and activity limitations of post-stroke that predict social participation: a systematic review. Top Stroke Rehabil. (2023) 30:589–602. doi: 10.1080/10749357.2022.2095086. PMID: 35787246

[65] Shipley J Luker J Thijs V Bernhardt J . The personal and social experiences of community-dwelling younger adults after stroke in Australia: a qualitative interview study. BMJ Open. (2018) 8:e023525. doi: 10.1136/bmjopen-2018-023525. PMID: 30559157 PMC6303598

[66] Wan X Sheung Chan DN Chun Chau JP Zhang Y Gu Z Xu L . Social participation challenges and facilitators among Chinese stroke survivors: a qualitative descriptive study. BMC Public Health. (2025) 25:468. doi: 10.1186/s12889-025-21592-z, PMID: 39910515 PMC11800553

[67] Goh HT Tan MP Mazlan M Abdul-Latif L Subramaniam P . Social participation determines quality of life among urban-dwelling older adults with stroke in a developing country. J Geriatr Phys Ther. (2019) 42:E77–e84. doi: 10.1519/jpt.0000000000000196. PMID: 29851747

[68] Zhou X Wang Y Zhou L . Social participation, resilience, and coping tendency in a sample of stroke survivors: a multi-center cross-sectional study in China. J Rehabil Med. (2024) 56:jrm12448. doi: 10.2340/jrm.v56.12448. PMID: 38175146 PMC10785685

[69] Zhang Y Jin Q Ji C Puan Y Chen L . Innovative Telerehabilitation Enhanced Care Program (ITECP) in young and middle-aged patients with hemorrhagic stroke to improve exercise adherence: protocol of a multicenter randomized controlled trial. BMJ Open. (2023) 13:e072268. doi: 10.1136/bmjopen-2023-072268. PMID: 38135318 PMC10749011

[70] Li Y Zhang W Ye M Zhou L . Perceived participation and autonomy post-stroke and associated factors: An explorative cross-sectional study. J Adv Nurs. (2021) 77:1293–303. doi: 10.1111/jan.14670. PMID: 33249635

[71] He YN Qin XH Lv JH Guo YP . Impact on participation and autonomy questionnaire (IPA): reliability and validity of the chinese version for stroke survivors. Int J Gen Med. (2025) 18:1721–9. doi: 10.2147/ijgm.S506798. PMID: 40161456 PMC11955180

[72] Fallahpour M Tham K Joghataei MT Jonsson H . Perceived participation and autonomy: aspects of functioning and contextual factors predicting participation after stroke. J Rehabil Med. (2011) 43:388–97. doi: 10.2340/16501977-0789. PMID: 21448554

[73] Mahmood A Deshmukh A Natarajan M Marsden D Vyslysel G Padickaparambil S . Development of strategies to support home-based exercise adherence after stroke: a Delphi consensus. BMJ Open. (2022) 12:e055946. doi: 10.1136/bmjopen-2021-055946. PMID: 34992120 PMC8739434

[74] Xu L Dong Q Jin A Zeng S Wang K Yang X . Experience of financial toxicity and coping strategies in young and middle-aged patients with stroke: a qualitative study. BMC Health Serv Res. (2024) 24:94. doi: 10.1186/s12913-023-10457-z. PMID: 38233772 PMC10795406

[75] Kuo WY Chen KL Tseng SM Chen CY . The care needs, preferences, and coping strategies of young stroke survivors: A qualitative study. J Cardiovasc Nurs. (2025). doi: 10.1097/jcn.0000000000001193. PMID: 40079548

